# Dietary Diversity and Its Contribution in the Etiology of Maternal Anemia in Conflict Hit Mount Cameroon Area: A Cross-Sectional Study

**DOI:** 10.3389/fnut.2020.625178

**Published:** 2021-02-04

**Authors:** Vanessa Tita Jugha, Judith Kuoh Anchang-Kimbi, Juliana Adjem Anchang, Kennedy Aminde Mbeng, Helen Kuokuo Kimbi

**Affiliations:** ^1^Department of Zoology and Animal Physiology, University of Buea, Buea, Cameroon; ^2^Pan African Institute for Development - West Africa, Buea, Cameroon; ^3^Medico-Sanitary Engineer Sub-Divisional Hospital Mutengene, Mutengene, Cameroon; ^4^Department of Medical Laboratory Sciences, University of Bamenda, Bamenda, Cameroon

**Keywords:** dietary diversity, anemia, pregnant women, Mount Cameroon area, cross-sectional study

## Abstract

**Background:** In the Mount Cameroon area, maternal anemia remains a major public health concern. We hypothesized that nutrient inadequacy may account for the level of anemia in pregnancy. Thus, this study examined the relative effect of dietary diversity on maternal anemia in the study area.

**Methods:** A total of 1,014 consenting pregnant women were enrolled in this cross-sectional study. Information on socio-demographic, antenatal characteristics, malaria and anemia control measures were documented. Dietary diversity (DD) was evaluated using the 24-h recall method and hemoglobin (Hb) levels (g/dl) measured using a portable Hb meter. Malaria parasitaemia was diagnosed by blood microscopy. Anemia status was trimester specific. Logistic regression analysis was used determine predictors of maternal anemia.

**Results:** Among the pregnant women enrolled, the mean DD score was 3.5±0.8 SD and only 10.4% had adequate dietary diversity. Anemia prevalence was 40.9%. Majority of the women consumed starchy staples (99.3%) while least consumed foods were dairy (4.5%), eggs (8.3%), fruits and vegetable (vitamin A-rich) (8.6%). A significant lower prevalence of anemia was associated with intake of dairy (*P* < 0.001), animal protein (*P* = 0.006), vitamin A-rich fruits and vegetables (*P* < 0.001). Furthermore, mean Hb levels were higher (*P* < 0.001) among women with diverse diets (12.39 ± 1.34) than in those with less diverse diets (10.85 ± 1.33). Predictors of anemia were as follows: study setting [Odd Ratio (OR) = 1.4, 95% CI: 1.07-1.94], occupation (OR = 1.9, 95% CI: 1.16-3.43), number of clinic visits (OR = 1.9, 95% CI: 1.27-2.91), trimester of pregnancy (OR = 3.2, 95% CI: 1.45-7.38), malaria parasitaemia (OR = 1.8, 95% CI: 1.33-2.68), out of home eating (OR = 1.4, 95% CI: 1.03-2.13), and DD (OR = 9.8, 95% CI: 4.56-20.80). The attributable risk of anemia due to dietary diversity was 82.9%.

**Conclusion:** In the study area, DD is a major risk factor for maternal anemia. This finding underscores the importance of content specific nutrition education during clinic visits to improve intake of protein and iron-rich food in anemia prevention.

## Introduction

Pregnancy is characterized by anatomical, physiological, and biochemical changes in the woman's body. These changes are accompanied by an increase in dietary energy and nutrient requirement to support these maternal adaptations during pregnancy as well as for nutrient delivery to the fetus. Thus, for adequate nutrient uptake, a pregnant woman's diet should composed of carbohydrates, protein, vitamins, minerals, and water (1). Notwithstanding, in low- and middle-income countries (LMICs) many women living in under resourced environments, suffer from undernutrition and pregnancy presents an additional burden on the women's nutritional requirement to meet the needs of both mother and the developing fetus. Consequences of inadequate nutrient intake in pregnancy include; low birth weight, intrauterine growth restriction as well as increased risk of maternal morbidity and mortality (2). More so, inadequate consumption of energy or specific nutrients during this “critical period” may contribute to deficits in the child's development as well as compromise survival later in adult life (3).

Dietary diversity is defined as increase in the variety of foods consumed over a specific reference period (4). A diverse diet not only ensures adequate intake of essential nutrients (5), it is known to boost the immune system thereby decreasing susceptibility to malaria parasitaemia in endemic areas (6, 7). Minimum dietary diversity for women (MDD-W) is a proxy indicator of dietary nutrient adequacy. This dichotomous indicator is the consumption of five or more food out of ten food groups (4, 8, 9). In LMICs, insufficient nutrient uptake and anemia among pregnant women are associated with monotonous starchy based diets with infrequent consumption of animal products and seasonal consumption of fruits and vegetables (8, 10). Foods rich in iron and vitamin A are known to reduce maternal anemia (11–13). Socioeconomic and cultural practices of the society in which individuals live may influence their dietary behavior (14). During antenatal care (ANC) clinics, pregnant women are educated on the health benefit of good nutrition practice in reducing anemia. Although each pregnant woman is given iron/folic acid (IFA) supplementation, they are encouraged to consume diverse food groups for adequate nutrient uptake to support pregnancy. However, this is not often the case, as financial constraints may delay early commencement of antenatal care and uptake of hematinics as well as hinder affordability of a diet rich in digestible forms of iron (15).

Globally, anemia is a major threat, affecting ~38.2 % of pregnant women (16) with those in LMICs accounting for 43% of the burden (17). Anemia causes are multi-factorial and contributing factors vary with geographical setting, season and dietary practice (18). A diet deficient in essential nutrients (iron, folate, vitamins A and B12) is the major cause of nutritional anemia (19) with the most prevalent being iron deficiency anemia (20). Hemoglobinopathies may increase risk of maternal anemia (21). Besides nutrient intake, other factors associated with anemia include maternal age, trimester of pregnancy (22), parity levels and ANC visit (23).

In Cameroon, antenatal care interventions such as health and nutrition-related education, screening and treatment of anemia, intermittent preventive treatment with sulphadoxine-pyrimethamine (IPTp-SP), IFA supplementation and distribution of insecticide treated nets (ITNs) have been intensified to reduce anemia and malaria in pregnancy. In spite of these interventions, anemia severity (41–53.3%) has not significantly decreased over the years in semi-urban and urbanized towns in the Mount Cameroon area (24, 25). Findings from a baseline study in the area suggest nutrient deficiency as a major contributor of anemia among pregnant women (26). Thus, we hypothesized that inadequate dietary intake may play a critical role in anemia in our setting. In this study, we described diet diversity characteristics and evaluated its contribution to anemia among pregnant women reporting for antenatal care in some medical facilities located in semi-urban and urbanized settings in the Mount Cameroon area. Findings of this study will identify contributory factors of maternal anemia and hence the designing of sustainable intervention strategies targeted at reducing anemia in pregnancy.

## Materials and Methods

### Study Area

The study was carried out at ANC units in four medical facilities from two health districts: Tiko health district; Tiko Holforth health center (THHC) and Mutengene Medical Center (MMC) and Buea Health District; Buea Integrated Health Center (BIHC) and Mount Mary Hospital (MMH). These antenatal clinics were selected based on the type of locality; semi-urban (Mutengene and Tiko) and urbanized (Buea) settings.

Tiko is a semi-urban settlement located at 18 to 80 m above sea level (asl) between latitude 009°21′57.3″N and longitude 04°04′22.0″E with a population size of 147,423 inhabitants (27). The town has daily mean temperature ranging between 28° and 33°C, a relative humidity of 83.1% and an mean rainfall of 4,524 mm (28). This area is characterized by the presence of a local seaport that allows for fishing, import, and export of goods between neighboring countries. The rich volcanic soil encourages farming activities and industrial agriculture. This Health District hosts the Cameroon Development Corporation (CDC) plantations where banana, rubber and oil palm are cultivated and exported.

Mutengene is a road junction, semi-urban town located at 240 meters above sea level (asl) between latitude 009°18′52.8″N and longitude 04°05′37.2″E with 40,000 inhabitants. Farming and business constitute the mainstay of the town. The mean temperature and relative humidity are 25.08°C and 83%, respectively [Cameroon Development Corporation (CDC) weather record].

Buea is an urbanized town located at 896 m asl between latitude 4°9′9.72″N and longitude 9°14′27.6″E with a population of 43,000 inhabitants. Buea is characterized by mean temperature range of 18–27°C, relative humidity of 80% and rainfall of 4,000 mm (29). Common occupational activities in this area include teaching in the public and private educational institutions, civil servants, and business owners (30).

The Mount Cameroon area has two distinct seasons: a rainy season, which spans from March to October with maximum rainfall (2000–10,000 mm) in August and September. The dry season lasts for 4 months (November-February) (31). *Plasmodium falciparum* is the major plasmodium species in the area (32, 33).

THHC, MMC, BIHC and MMH offer services for antenatal care, preventive, curative, and delivery at low costs (34). In the study area, nearly all pregnant women attend ANC clinic at least once, but only 62% complete the recommended four or more ANC visits, with majority (73%) registering for ANC initiation in the second trimester of pregnancy (25). Pregnant women living in the Mount Cameroon area are commonly exposed to *P. falciparum* infection (peripheral blood infection) with prevalence range of 13.4–22.4% (24, 35). In Cameroon, the transition from a two to three doses of IPTp-SP policy took place in 2012 (36). Recent findings revealed a significant improvement in the uptake of ≥3 doses of SP in this area with a coverage of 47% (25). Additional benefits of repeated doses of SP in combination with ITNs use in reducing peripheral malaria parasitaemia and maternal anemia have been reported in the area (24). Though malaria-related anemia has decreased over the years, about 75% of the anemic cases are non-malaria related (26).

### Sampling Design, Population, and Sampling Procedure

A descriptive and analytical cross-sectional study comprising of pregnant women attending ANC visit in four health facilities in semi-urban and urbanized settings in the Mount Cameroon area was carried out from July, 2018 to September, 2019.

The population sample size was determined using the Cochrane formulae for cross-sectional studies (37)

$$\begin{array}{l} N\;=\;\frac{Z^{2}P\left(1-P\right)}{d^{2}} \end{array}$$

The sample size N was calculated based on the maternal anemia prevalence (p) of ~40% (24), a margin of error of 5%, 95% confidence interval (CI), and an attrition rate of 10%. The minimum sample size per health district was 408. A non-probability sampling technique was used to enroll consenting pregnant women between the ages 15–49 years reporting for ANC initiation or follow-up. Recruitment involved approaching participants who were available (convenience) and after informing them of the study, those who met the eligibility criteria and gave their consent were enrolled consecutively. Pregnant women with reported history of hypertensive disorders, diabetes, or preeclampsia were excluded from the study.

### Questionnaire Survey

An interview-guided structured questionnaire was used to record information pertaining to maternal socio-demographic characteristics, obstetric data, IPTp-SP, IFA uptake, ITN usage, medical history as well as dietary intake 24-h prior to survey. Adequate IPTp-SP uptake was defined as uptake of at least 3 or more SP doses as stipulated by the World Health Organization (36). Complete ANC attendance was defined as four or more clinic visits during pregnancy (38). The questionnaire on MDD-W guided obtention of data on dietary diversity (4).

### Dietary Assessment

Minimum dietary diversity was assessed qualitatively using the 24-h recall method. This reference period was considered appropriate because it is less prone to recall error, less cumbersome for the respondents and corresponds with the time period used in several studies on DD (39). Each study participant was asked to list and describe the exact composition of all food items and drinks consumed (day or night) prior to survey (40). Foods consumed out of the home during the specified period were assessed.

Dietary diversity scores were estimated using 10 food groups which include; (1) grains, white roots, tubers and plantains (starchy staples), (2) pulses (beans, peas and lentils), (3) nuts and seeds, (4) dairy products, (5) meat, poultry and fish, (6) eggs, (7) dark green leafy vegetables, (8) vitamin A-rich food group (fruits and vegetables), (9) vegetables and (10) fruits. Consumption of food within any food group, equivalent to one tablespoon, was assigned a score (4, 39). Furthermore, the total score per participant was calculated as the sum total of the scores for the different food groups consumed (41). Women who consumed < 5 food groups were considered to have a poor DD (score range: 1–4) and those who consumed at least 5 food groups were considered to have a good DD (score range: 5–10) (4).

### Sample Collection and Laboratory Analysis

Venous blood sample (2 ml) was collected from each pregnant woman by a licensed laboratory technician using standard procedures. Hemoglobin (Hb) levels were determined using a portable Urit12® Hb meter (URIT Medical Electronics Co., Ltd. Guangxi, China) and values documented to the nearest 0.1 g/dl. Anemia status was trimester specific and defined as follows; Hb <11.0 g/dl for women in the first (1–13 weeks of gestation) and third (≥27 weeks of gestation) trimesters and Hb <10.5 g/dl for those in the second trimester (14–26 weeks of gestation) (38, 42, 43). Blood films were prepared for the detection of malaria parasitaemia (44). Briefly, two drops of whole blood were placed on a labeled glass slide, thick and thin blood smears were prepared and air-dried. The thin blood films were fixed in absolute methanol for 30 s and later both films were stained with 10% Giemsa for 15 min and examined under a light microscope by two independent microscopists. A smear was declared positive for malaria parasitaemia if any asexual forms of *P. falciparum* were identified after examining 100 high power fields (44).

### Ethical Considerations

The study protocol was reviewed and approved by the Institutional Review Board of the University of Buea (Ref No: 2019/967-05/UB/SG/IRB/FHS). Administrative authorization was obtained from the South West Regional Delegation of Public Health, Buea. Participation in the study was voluntary and individuals who gave their consent signed a written consent form.

### Data Analysis

Data was analyzed using IBM SPSS statistics software version 20. For continuous variables, the Kolmogorov-Smirnov test was used to check for normality and variables summarized into mean and standard deviation (SD). Proportions were used to describe categorical variables. Student *t*-test was used to compare the mean Hb levels per MDD-W categories. Pearson Chi-square test (χ^2^) was used to evaluate differences in proportions.

The main outcome (dependent) variable was anemia. Covariates (independent variables) included; maternal age, setting, marital status, occupation, educational level, climatic season of enrolment, gravidity status, trimester of pregnancy, number of ANC visits, malaria parasite status, SP doses, ITN usage, out of home eating, and MDD-W. Prior to multivariable regression analysis, confounders were checked for collinearity by examining the variance inflation factor (VIF). Multicollinearity was absent with all covariates having a VIF < 2. Binary logistic regression model (enter method) was run to examine the independent effect of covariates on anemia status. All 1,014 participants were included in the model. The attributable risk (AR%) of anemia due to dietary diversity was calculated using an established method (45). Significant levels were set at 95% confidence interval (CI); *P* < 0.05.

## Results

### Characteristics of the Study Population

Of the 1,014 consenting pregnant women enrolled, 79 were from THHC, 430 from MMC, 163 from MMH and 342 from BIHC. In general, 50.2 and 49.8% of the participants were from Tiko health district (THD) and Buea health district (BHD), respectively. With respect to gestational age, majority of these women were in their second (41.2%) and third (55.4%) trimesters of pregnancy (Table 1). Mean (±SD) maternal age was 26.7 ± 5.48 (range;15-46 years). Trading (business) was the most common occupational activity (43.6%), while only 14.6% were civil servants. About 42% were housewives, farmers, or students. More than half (62.2%) of the women were married. The highest level of education attained by most (53.1%) of the women was secondary education. Unexpectedly, majority of women attended < 4 ANC visits (77.3%) and only 12.2% had adequate IPTp-SP uptake (≥3 SP doses). Maternal age, educational level, occupational status, gravidity, trimester of pregnancy, IPTp-SP uptake and ITN usage varied between the two health districts. Meanwhile, marital status, ANC attendance and malaria parasite status were comparable between health districts (Table 1).

**Table 1:** Baseline characteristics of the study population per study setting.

**Variables**	**Categories**	**THD % (*n*)**	**BHD % (*n*)**	**Total % (*N*)**	***P* value**
Age (15-46 years)	≤20	19.6 (100)	8.9 (45)	14.3 (145)	<0.001
	21-25	29.7 (151)	28.5 (144)	29.7 (295)	
	>25	50.7 (258)	62.6 (316)	56.6 (574)	
Marital Status	Single	39.7 (202)	35.8 (181)	37.8 (383)	0.207
	Married	60.3 (307)	64.2(324)	62.2 (631)	
Educational level	Primary	28.3 (144)	11.9 (60)	20.1 (204)	<0.001
	Secondary	58.0 (295)	48.1 (243)	53.1 (538)	
	Tertiary	13.8 (70)	40.0 (202)	26.8 (272)	
Occupation status	Housewife	18.7 (95)	14.3 (72)	16.5 (167)	<0.001
	Farmer	9.4 (48)	2.2 (11)	5.8 (59)	
	Business	47.2 (240)	40.0 (202)	43.6 (442)	
	Students	14.9 (76)	24.2 (122)	19.5 (198)	
	Civil servants	9.8 (50)	19.4 (98)	14.6 (14.8)	
No of ANC	<4 clinic visits	77.6 (395)	77.0 (389)	77.3 (784)	0.827
	≥4 clinic visits	22.4 (114)	23.0 (116)	22.7 (230)	
Gravidity	Primigravid	31.2 (159)	38.0 (192)	34.6 (351)	0.047
	Secundigravid	25.3 (129)	25.1 (127)	25.2 (256)	
	Multigravid	43.4 (221)	36.8 (186)	40.1 (407)	
Trimester of pregnancy	First trimester	2.0 (10)	5.0 (25)	3.5 (35)	0.033
	Second trimester	41.7 (212)	40.8 (206)	41.2 (418)	
	Third trimester	56.4 (287)	54.3 (274)	55.3 (561)	
IPTp-SP dose uptake	1 dose	64.6 (329)	70.7 (357)	67.7 (686)	0.003
	2 doses	19.6 (100)	20.6 (104)	20.1 (204)	
	≥3 doses	15.7 (80)	8.7 (44)	12.2 (124)	
Women who own ITN	No	28.7 (146)	28.9 (146)	28.8 (292)	0.936
	Yes	71.3 (363)	71.1 (359)	71.2 (722)	
ITN usage	No	53.2 (271)	68.7 (347)	60.9 (618)	<0.001
	Yes	46.8 (238)	31.3 (158)	39.1 (396)	
IFA uptake	No	29.5 (150)	27.5 (139)	28.5 (289)	0.493
	Yes	70.5 (359)	72.5 (366)	71.5 (725)	
Malaria status	Negative	81.9 (417)	82.6 (417)	82.2 (834)	0.787
	Positive	18.1 (92)	17.4 (88)	17.8 (180)	
MDD-W	< 5 food groups	91.0 (463)	88.3 (446)	89.6 (909)	0.167
	≥ 5 food groups	9.0 (46)	11.7 (59)	10.4 (105)	

*ANC, antenatal clinic; IPTp-SP, intermittent preventive treatment in pregnancy with sulfadoxine-pyrimethamine; ITN, insecticide treated net; IFA, iron folic acid; MDD-W, minimum dietary diversity for women of reproductive age*.

### Dietary Diversity

The mean (±SD) MDD score of the study population was 3.57± 0.82 (score range: 1–7). Of the 1,014 women, 10.4% (95% CI: 8.6–12.4; *n* = 105) met the FAO criteria for MDD-W, whereas, 89.6% (95% CI: 87.7-91.4; *n* = 909) of the participants consumed diets with < 5 food groups. Considering the different foods consumed 24-h prior to study, nearly all women (99.3%) ate starchy staples, more than three-quarters (86.2%) consumed animal products (meat, poultry, fish), two-third (67.5%) of the women consumed other vegetables. Green leafy vegetables, fruits, pulses, nuts and seeds were consumed by 29.5, 21.4, 17.9, and 13.7% of the participants, respectively. Intake of dairy products (4.5%), eggs (8.3%), and vitamin A-rich fruits and vegetables (8.6%) was minimal (Table 2).

**Table 2:** Proportion of the different food groups consumed by the study participants 24-h prior to survey.

**Food groups**	**Frequency (*n*)**	**Percentage (%)**
Grains, white roots, tubers and plantains	1007	99.3
Pulses (beans, peas and lentils)	181	17.9
Nuts and seeds	139	13.7
Meat, poultry and fish (iron rich foods)	874	86.2
Dairy products	46	4.5
Eggs	84	8.3
Dark green leafy vegetables	299	29.5
Other vitamin A-rich fruits and vegetables	87	8.6
Other vegetables	684	67.5
Other fruits	217	21.4

### Prevalence of Maternal Anemia

**Figure 1:**
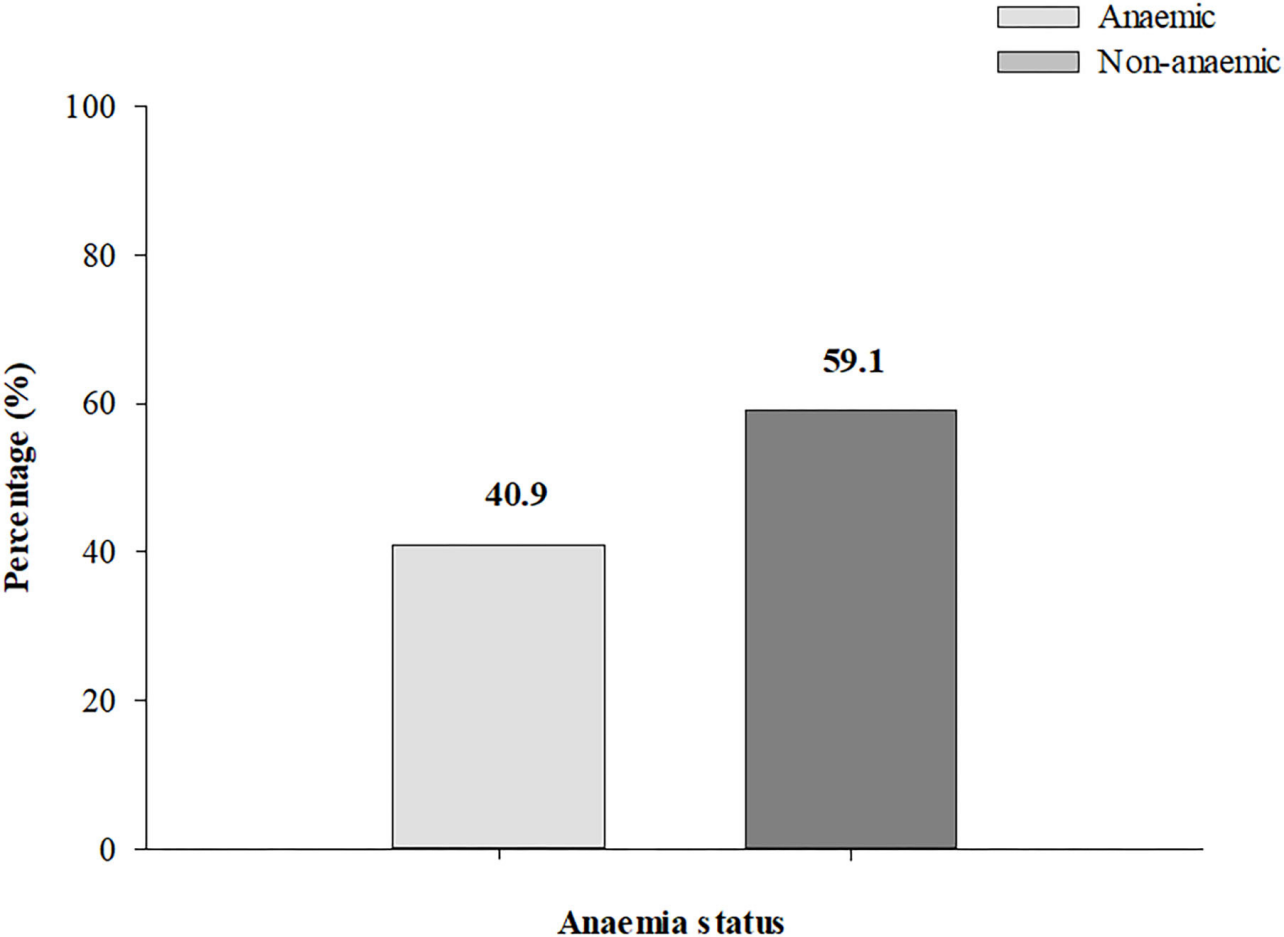
Prevalence of anemia among pregnant women.

Mean Hb concentration among the women in the first, second and third trimester was 11.43 ± 1.50 g/dl, 10.95 ± 1.50 g/dl, and 11.03 ± 1.33 g/dl, respectively. The overall prevalence of anemia was 40.9% (95% CI: 37.9-44.0; *n* = 415) (Figure 1).

### Association Between Dietary Diversity, Maternal Anemia Status, and Hemoglobin Levels

**Figure 2:**
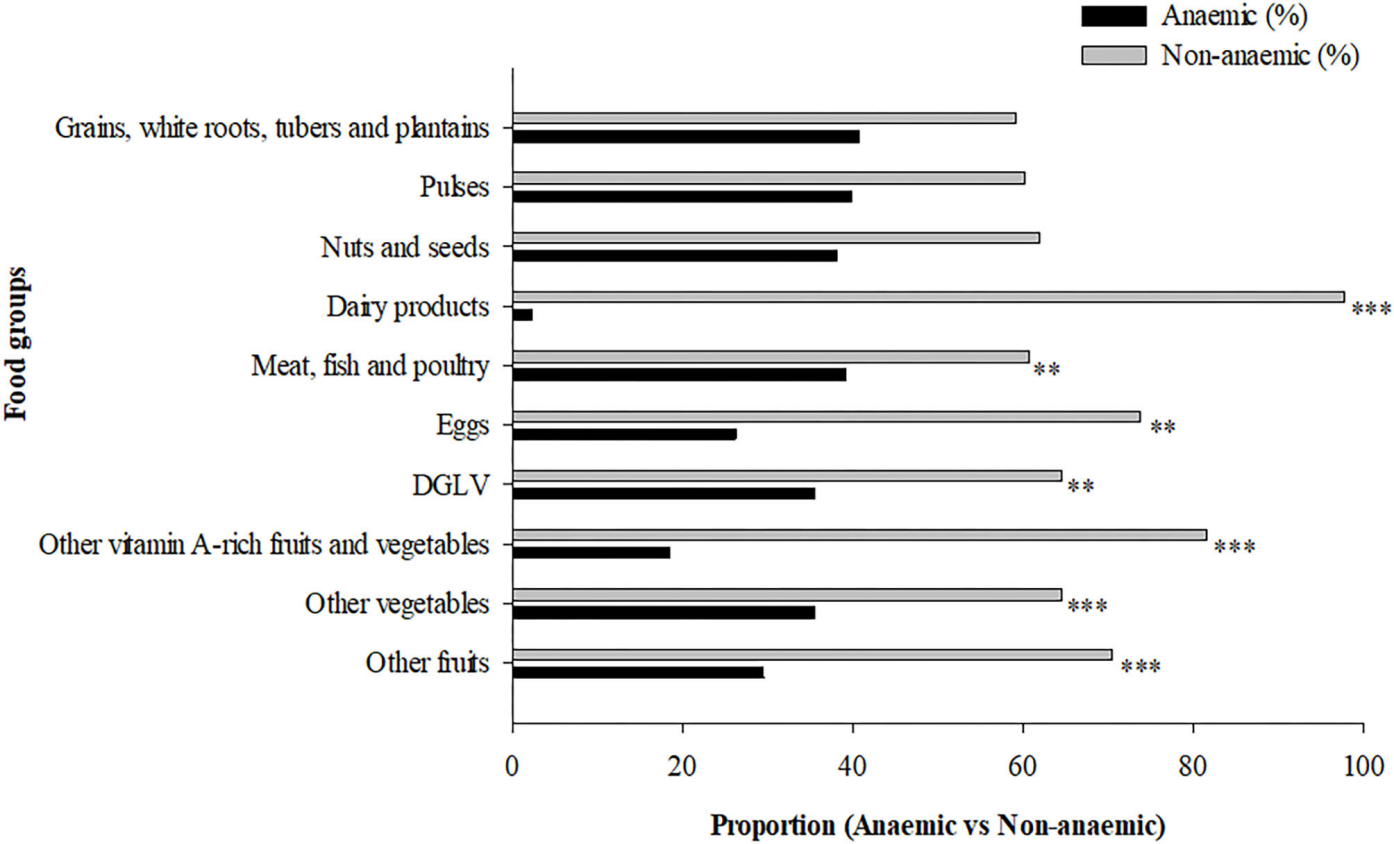
Maternal dietary diversity during pregnancy and anemia status. **P < 0.01, ***P < 0.001, Pearson Chi-square test. DGLV, dark green leafy vegetable.

The distribution of respondents according to the different food groups in relation to their anemic status are presented in Figure 2. Intake of dairy products (*P* < 0.001), meat, fish and poultry (*P* = 0.006), eggs (*P* = 0.004), dark green leafy vegetables (*P* = 0.022), vitamin A-rich fruits and vegetables (*P* < 0.001), consumption of vegetables (*P* < 0.001), fruits (*P* < 0.001) was significantly associated with lower prevalence of anemia (Figure 2).

**Figure 3:**
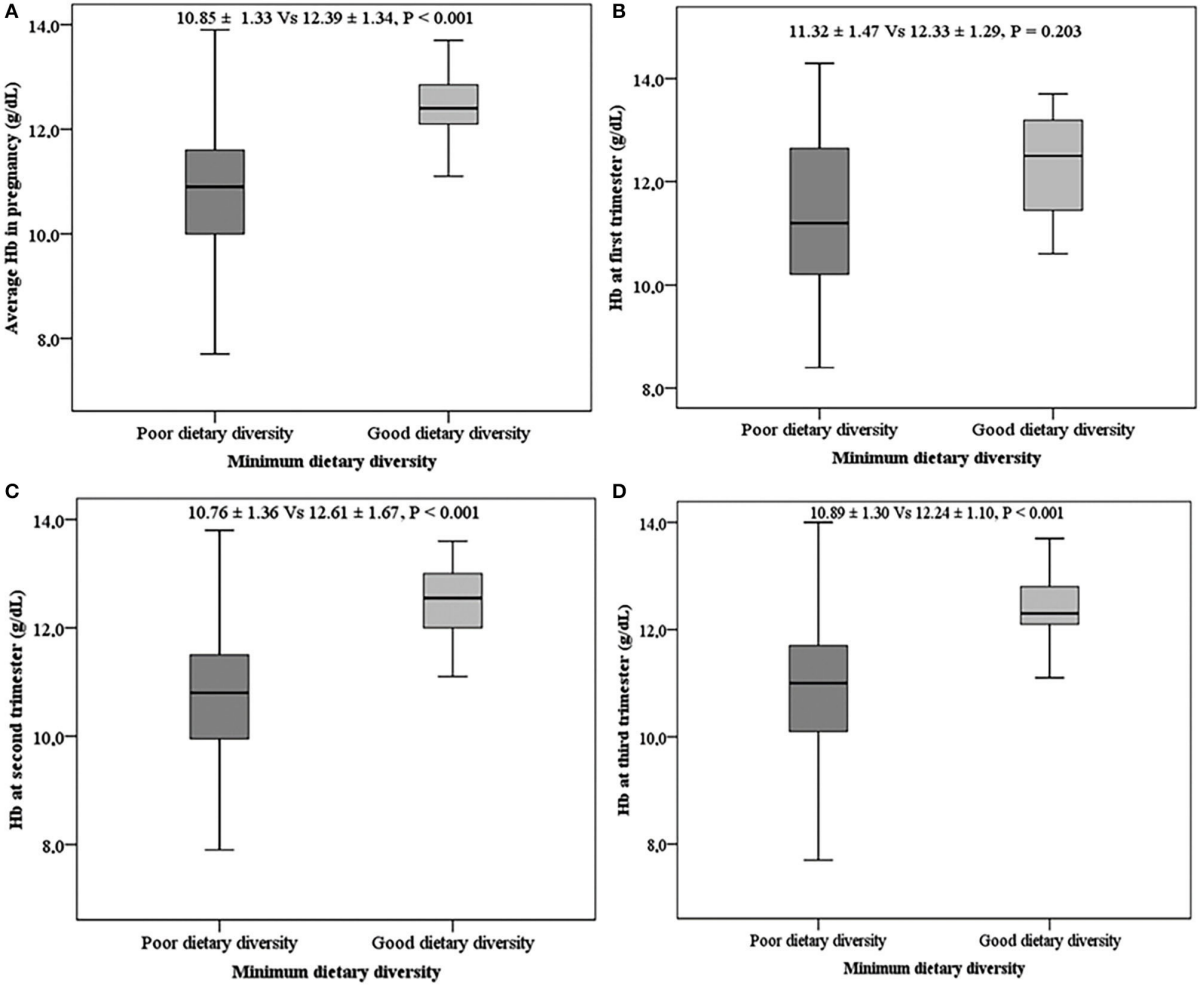
Mean hemoglobin levels with respect to dietary diversity. **(A)** Mean Hb during pregnancy, **(B)** mean Hb in the first trimester, **(C)** mean Hb in the second trimester, **(D)** mean Hb in the third trimester.

Overall, the mean Hb levels were higher (*P* < 0.001) among women with good DD (12.39 ± 1.34) when compared with those with poor DD (10.85 ± 1.33). This observation was similar across the different trimesters of pregnancy except for the first trimester of pregnancy (Figure 3).

### Factors Associated With Maternal Anemia

Binary logistic regression was performed to ascertain the determinants associated with the risk of maternal anemia. The predictor variables of anemic status were; study setting, level of education, occupation status, number of ANC clinic visits, trimester of pregnancy, malaria parasitaemia, MDD-W, and out of home eating (Table 3). Considering the odd ratios, BHD (OR = 1.4, 95% CI: 1.07-1.94), secondary level of education (OR = 1.5, 95% CI: 1.07-2.31), civil service status (OR = 1.9, 95% CI: 1.16-3.43), <4 ANC visits (OR = 1.9, 95% CI: 1.27-2.91), third trimester of pregnancy (OR = 3.2, 95% CI: 1.45-7.38), malaria parasitaemia (OR = 1.8, 95% CI: 1.33-2.68), poor DD (OR = 9.8, 95% CI: 4.65-20.80), and out of home eating (OR = 1.4, 95% CI: 1.03-2.13) were associated with increased odds of anemia compared with their respective counterparts (Table 3). In addition, the attributable risk (AR%) of maternal anemia due to dietary diversity was 82.9% (95% CI: 60.8-105.2).

**Table 3:** Factors associated with maternal anemia.

**Factors**	**Categories**	**Total *N***	**Anemic % (*n*)**	**COR (95% CI)**	***P* value**	**AOR (95%CI)**	***P* value**
Climatic seasons	Dry	213	46.0 (98)	1.3 (0.95-1.76)	0.090	1.2 (0.96-1.75)	0.169
	Rainy	801	39.6 (317)	REF		REF	
Health district	THD	509	38.7 (197)	REF	0.148	REF	
	BHD	505	43.2 (218)	1.2 (0.93-1.54)		1.4 (1.07-1.94)	0.015
Age (15-46 years)	≤20	145	45.5 (66)	1.2 (0.86-1.80)	0.477	1.4 (0.82-2.41)	0.215
	21-25	295	40.3 (119)	1.0 (0.75-1.34)		1.1 (0.79-1.66)	0.447
	>25	574	40.1 (230)	REF		REF	
Marital status	Single	383	39.2 (150)	REF	0.374	REF	
	Married	631	42.0 (265)	1.1 (0.86-1.45)		1.1 (0.87-1.61)	0.269
Education level	Primary	204	38.7 (79)	1.1 (0.79-1.68)	0.030	1.1 (0.68-1.91)	0.594
	Secondary	538	44.6 (240)	1.4 (1.09-1.99)		1.5 (1.07-2.31)	0.020
	Tertiary	272	35.3 (96)	REF		REF	
Occupation status	Housewives	167	40.1 (67)	1.1 (0.75-1.75)		1.2 (0.75-1.98)	0.422
	Farmers	59	44.1 (26)	1.3 (0.74-2.43)		1.3 (0.63-2.78)	0.454
	Business	442	41.4 (183)	1.2 (0.85-1.70)	0.645	1.2 (0.77-1.87)	0.417
	Civil servants	198	44.6 (66)	1.3 (0.89-2.12)		1.9 (1.16-3.43)	0.012
	Students	148	36.9 (73)	REF		REF	
ANC attendance	<4 clinic visits	784	43.8 (343)	1.7 (1.24-2.33)	0.001	1.9 (1.27-2.91)	0.002
	≥4 clinic visits	230	31.3 (72)	REF		REF	
Gravidity	Primigravid	351	39.3 (138)	0.8 (0.62-1.12)		0.8 (0.56-1.34)	0.530
	Secundigravid	256	39.1 (100)	0.8 (0.60-1.14)	0.397	0.8 (0.58-1.24)	0.407
	Multigravid	407	43.5 (177)	REF		REF	
Trimester of pregnancy	First trimester	35	31.4 (11)	REF		REF	
	Second trimester	418	36.6 (153)	1.2 (0.60-2.64)	0.019	1.4 (0.64-3.07)	0.396
	Third trimester	561	44.7 (251)	1.7 (0.84-3.67)		3.2 (1.45-7.38)	0.004
IPTp-SP uptake	First dose	686	42.6 (292)	1.5 (1.03-2.33)		1.5 (0.89-2.57)	0.121
	Second dose	204	40.7 (83)	1.4 (0.90-2.30)	0.099	1.2 (0.73-2.09)	0.420
	≥ three doses	124	32.3 (40)	REF		REF	
ITN usage	Yes	396	40.9 (162)	REF	0.993	REF	
	No	618	40.9 (253)	1.0 (0.77-1.29)		0.9 (0.73-1.29)	0.849
IFA uptake	Yes	725	40.8 (296)	REF	0.919	REF	
	No	289	41.2 (119)	1.0 (0.76-1.33)		1.1 (0.81-1.63)	0.408
Malaria status	Positive	180	53.9 (97)	1.8 (1.37-2.62)	0.000	1.8 (1.33-2.68)	<0.001
	Negative	834	38.1 (318)	REF		REF	
MDD-W	<5 food groups	909	44.8 (407)	9.8 (4.72-20.45)		9.8 (4.56-20.80)	<0.001
	≥5 food groups	105	7.6 (8)	REF	0.000	REF	
Out of home eating	Yes	173	46.8 (81)	1.3 (0.96-1.85)	0.083	1.4 (1.03-2.13)	0.032
	No	841	39.7 (334)	REF		REF	

*ANC, antenatal clinic; IPTp-SP, intermittent preventive treatment in pregnancy with sulfadoxine-pyrimethamine; ITN, insecticide treated net; IFA, iron folic acid uptake; MDD-W, minimum dietary diversity for women; COR crude odd ratio; AOR adjusted odd ratio*.

## Discussion

The causes of anemia are multi-factorial and the contribution of each of the factors may vary with dietary practice, geographical setting, sociodemographic, and season. This study determined the dietary diversity status of pregnant women and assessed its attribution in maternal anemia in the Mount Cameroon area. Extensive studies on DD has been conducted in several African settings, however, this is the first report on DD and its association with anemia in pregnant Cameroonian women.

The mean DD score of the pregnant women in this study was 3.5 ± 0.8 SD and only 10.4% of the pregnant women had adequate dietary diversity. Most (89.6%) of the pregnant women were not consuming adequate food (below the MDD-W score recommended for pregnant women) (4). A similar finding has been reported from Oromia region, Central Ethiopia (46). Contrarily, the mean DDS in this study is less than values reported by Yeneabat et al. (47) in Northwest Ethiopia and Ayensu et al. (48) in rural and urban areas of Ghana. These variations may be attributed to socio-demographic, socio-economic, geographical, and seasonal differences (46). Higher dietary diversity or variety of foods ensures adequate intake of essential nutrients that improves the probability of good health (49). In contrast with the present study, the pregnant women consumed less than the expected classes of food, which may be linked to micronutrient deficiencies.

In this study, the diet of pregnant women in the previous 24 h was composed mainly of starchy food group (grains, white roots, tubers and plantains) (99.3%), meat, poultry and fish group (86.2%), and vegetables (67.5%). Conversely, dairy products, eggs, fruits, and vegetables (vitamin A-rich) were least consumed foods. Comparable findings have been reported by authors in Tanzania (50), Ethiopia (46), and Kenya (51). The low socio-economic status of women resident in the study area may be associated with the inability to afford animal products. Moreover, many women in this study setting, on average, rely on a monthly income of <30.000 FCFA (franc des Communautés Financières d'Afrique) (~60 USD) (35). Also, pregnant women may have missed scheduled ANC opportunities to be sensitized or educated on healthy lifestyle and fight against malnutrition (52). It worth noting that, more than 75% of the pregnant women attended <4 ANC visits. In Cameroon, averagely 65% of women in each time period receives antenatal care four or more times from any health provider (53). Since 2017, the Anglophone crisis in the English-speaking regions of Cameroon has affected particularly rural and peri-urban communities. These areas have been hit hardest by violence and its indigenes have become internally displaced and also making it difficult for pregnant women to attend ANC clinic in some of these areas.

Our findings confirm that anemia is a severe public health problem (40.9%) in this region of the country (16). The level of anemia revealed by this study is comparable with the national prevalence (39.7%) (range: 23.7–53.9) (53) but higher than the global average of 38.2% (16). On the other hand, the finding of this study is below the prevalence of anemia (52.5%) in the Northern region of Cameroon (54) and in Ghana (56.5%) (48). This demonstrates variation in the prevalence of anemia within different settings in the same country. This may be attributed specifically to geographical variations, differences in socio-economic status, cultural and dietary patterns (55).

The results of this study showed that study setting, maternal educational level, occupation status, number of ANC visits, gestational age, malaria parasitaemia, DD, and out of home eating were predictors of maternal anemia. These identified factors are discussed as follows.

Although DD is not the only contributing factor of anemia in pregnancy, the present study revealed that DD is the most pressing constraint. Low DD score (<5 food groups) increased the odds of anemia by ~10 folds when compared with high DD score (≥5 food groups). In addition, DD had a positive effect on Hb levels regardless of the gestational age. More so, more than 80% of anemia was attributable to dietary diversity. These findings corroborate studies by Lebso et al. (13) and Delil et al. (56) in Southern Ethiopia. However, in Northern Ghana, diet was not one of the protective factors against anemia (57). Suboptimal dietary intake increases the risk of anemia which has negative effects on the growth and development of the fetus (21, 58) and lactation (57). A diverse diet composed of different food groups rich in micronutrients particularly, iron, vitamin A, vitamin B_12_, and folic acid functions to promote red blood cell production thus preventing anemia among women of reproductive age (59, 60). This study demonstrated that women who ate out of their homes were 1.4 times more likely to be anemic. Eating out of home is associated with poor dietary quality and a predisposing factor for higher energy and fats relative to lower micronutrient intake (61). In addition, out of home foods are limited in essential nutrients especially, vitamin C, calcium, and iron (57, 62, 63).

It is well established that malaria parasitaemia causes and/or aggravates anemia (31, 64, 65). The occurrence of malarial anemia (23.4%) among pregnant women is not an uncommon finding in the Mount Cameroon Area (26, 31). Although, *P. falciparum* infection plays a role in the pathophysiology of anemia, however, it is not the major contributing factor of anemia in our endemic setting.

Our results showed that women who attended ANC clinic less than four times were more likely (OR = 1.9) to be anemic. Regular ANC visits permits increase uptake of prophylactic measures against malarial infection, iron and folic acid supplementation as well as enables the pregnant woman acquire knowledge on adequate nutrition and health education (57). The number of ANC visits was seen as a strong predictor of anemia among pregnant women in Ghana (57, 66). Pregnant women in their third trimester of gestation were 3.2 times more likely to be anemic than those in their first and second trimester. The association between gestational age and anemia agrees with findings by El Aishiry et al. (67) in Egypt, Lebso et al. (13) in Ethiopia and Wemakor (68) in Ghana. During the third trimester of gestation, the haemo-dilutional effect of pregnancy and increase in nutritional demand for the mother and growing fetus are maximal (69).

Socio-demographic factors associated with the occurrence of anemia included educational status, occupation and residence. Women engaged in civil service were 1.9 times at increased risk of anemia when compared with other occupations. This could be related to missed meals, since they are busy throughout the day. Similarly, severe anemia was significantly more prevalent among women in waged labor in rural Nepali (70). Several studies have reported reduced risk of anemia in association with higher level of education (56, 71–73). On the contrary, this study demonstrated otherwise. This could be explained partly by bias as more than 50% of pregnant women in the study population have attained a secondary level of education. Education enable women to adopt better health seeking behaviors as well as utilization of information that are important to nutritional status. Residents in BHD were 1.4 times more affected by anemia than those of THD. This difference may be related to variation in dietary micronutrient profile and socio-demographics of these women. THD is characterized by mainly farming activities, industrial agriculture and business whereas common occupational activities in BHD include teaching in the public and private educational institutions and civil servants and business. Less anemia among residents in THD than in BHD is likely linked to availability and consumption of variety of farm produce (rich in iron) in THD. Nevertheless, further studies on dietary iron intake, iron bioavailability and maternal anemia are crucial.

## Strengths and Limitations

This study confirms that anemia is an important public health problem among pregnant women in Africa and highlights the importance of simple preventive actions to increase dietary diversity by promoting nutritional education and the awareness about the importance of having a balanced diet for pregnant women. However, this study had some limitations. Only qualitative dietary assessment was done. Though quantitative methods are best for assessing nutrient adequacy, these methods are cumbersome and not affordable in limited resource settings like ours. Simple measures of dietary diversity such as MDD-W indicator, equally, has been shown to reflect micronutrient adequacy (4). We attest that temporality is the primary limitation of cross-sectional study design. However, we used both descriptive and analytical cross-sectional study designs, and sufficiently large sample size population.

## Conclusion

In general, 40.9% of the pregnant women had anemia. Only 10.4% had adequate dietary diversity while 89.6% did not meet the FAO indicator of minimum dietary diversity for women. This study revealed that diets of these women are composed mainly of starchy staples, but less of dairy products, eggs, dark green leafy vegetables, fruits, and vegetables (vitamin A-rich) as well as fruits. Intake of iron-rich diets improved Hb levels. The predictors of anemia were: setting, educational level, occupation, number of ANC attendance, trimester of pregnancy, malaria parasitaemia, MDD-W and eating out of home. Poor DD was a key contributor of anemia (AR% = 82.9%). Nutritional education on the importance of a balanced diverse diet should be intensified during ANC to curb the risk of anemia in pregnancy in the Mt. Cameroon area.

## Data Availability Statement

The raw data supporting the conclusions of this article will be made available by the authors, without undue reservation.

## Ethics Statement

This study, involving human participants, was reviewed, and approved by Institutional Review Board of the University of Buea. Informed consent to participate in this study was provided by the participants and/or legal guardian.

## Author Contributions

VJ and JA-K conceived and designed the study. VJ and KM conducted the research including data collection. VJ and JA were responsible for data management and analysis. VJ interpreted the data and wrote the first draft of the manuscript. JA-K supervised and critically revised the manuscript for important and intellectual content. HK supervised and revised the manuscript for important and intellectual content. All authors read and approved the final manuscript.

## Conflict of Interest

The authors declare that the research was conducted in the absence of any commercial or financial relationships that could be construed as a potential conflict of interest.
